# ALDH1: A potential therapeutic target for cancer stem cells in solid tumors

**DOI:** 10.3389/fonc.2022.1026278

**Published:** 2022-10-28

**Authors:** Yaolu Wei, Yan Li, Yenan Chen, Pei Liu, Sheng Huang, Yuping Zhang, Yanling Sun, Zhe Wu, Meichun Hu, Qian Wu, Hongnian Wu, Fuxing Liu, Tonghui She, Zhifeng Ning

**Affiliations:** ^1^ School of Basic Medicine Sciences, Xianning Medical College, Hubei University of Science and Technology, Xianning, China; ^2^ Xianning Medical College, Hubei University of Science and Technology, Xianning, China

**Keywords:** ALDH1, stem cell markers, solid tumors, targeted therapy, cancer resistance

## Abstract

Solid tumors can be divided into benign solid tumors and solid malignant tumors in the academic community, among which malignant solid tumors are called cancers. Cancer is the second leading cause of death in the world, and the global incidence of cancer is increasing yearly New cancer patients in China are always the first. After the concept of stem cells was introduced in the tumor community, the CSC markers represented by ALDH1 have been widely studied due to their strong CSC cell characteristics and potential to be the driving force of tumor metastasis. In the research results in the past five years, it has been found that ALDH1 is highly expressed in various solid cancers such as breast cancer, lung cancer, colorectal cancer, liver cancer, gastric cancer, cervical cancer, esophageal cancer, ovarian cancer, head,and neck cancer. ALDH1 can activate and transform various pathways (such as the USP28/MYC signaling pathway, ALDH1A1/HIF-1α/VEGF axis, wnt/β-catenin signaling pathway), as well as change the intracellular pH value to promote formation and maintenance, resulting in drug resistance in tumors. By targeting and inhibiting ALDH1 in tumor stem cells, it can enhance the sensitivity of drugs and inhibit the proliferation, differentiation, and metastasis of solid tumor stem cells to some extent. This review discusses the relationship and pathway of ALDH1 with various solid tumors. It proposes that ALDH1 may serve as a diagnosis and therapeutic target for CSC, providing new insights and new strategies for reliable tumor treatment.

## Introduction

According to global cancer data, 9.96 million people worldwide will die by 2020, of which China ranks first in the world in terms of cancer deaths (1). The cause of cancer death is still unclear and is currently mainly related to cancer stem cells and drug resistance. In recent years, with the introduction of the stem cell concept into cancer research, researchers have found that cancer heterogeneity is signficant source of disease progression and treatment failure, and cancer stem cells (CSCs) are the source of heterogeneity (2). Although the number is scarce, it has a solid carcinogenic, robust carcinogenic, carcinogenic solid ability and the potential to generate various types of cells that constitute tumors (3). The tumor is a stem cell disease and acetaldehyde dehydrogenase 1 (ALDH1) is one of the most essential markers in CSCs. The expression level of ALDH1 in solid tumor tissues is higher than in normal tissues. Therefore, in recent years, more and more researchers have studied the possibility of ALDH1 as a potential therapeutic target for CSCs.

ALDH1 is one of the aldehyde dehydrogenases, located mainly on chromosome 9q21 (4). As an isoenzyme of acetaldehyde dehydrogenase, ALDH1 exists mainly in the cytoplasm of liver cells. It is responsible for further oxidation of acetaldehyde as a substrate by alcohol dehydrogenase to harmless acetic acids. As a cellular lipase, ALDH1 plays a vital role in gene expression and tissue differentiation in many tissues. Current studies have found that ALDH1 is very likely to be a stem cell marker for various solid tumors (5–7). With in-depth study by researchers, ALDH1 is highly expressed in lung cancer (8), invasive cervical cancer (9), breast cancer (10), ovarian cancer (7), colorectal cancer (11), gastric cancer (5), esophageal cancer (12), head and neck cancer (13) and other solid cancers from clinical research. ALDH1A1 is the main component of ALDH1, and the activation of ALDH1 depends mainly on ALDH1A1. Recent studies have shown that the higher the level of ALDH1A1, the worse the prognosis for patients, especially for tumors of the digestive system. Furthermore, ALDH1 is not only involved in many critical biological functions such as cell differentiation and resistance to radiation therapy and chemotherapy but also clinical research found that high expression of the ALDH1 gene signature in cancer tissue is positively correlated with malignant progression in cancer patients (14). Therefore, it should be clear whether ALDH1 can be used as a therapeutic target for various solid tumor stem cells? When targeting ALDH1, can it inhibit the proliferation and differentiation of tumor stem cell markers and reduce the recurrence and metastasis of malignant tumors? Thus, it can provide a new target and basis for the treatment of solid tumors.

## ALDH1 and mechanism

### Mechanisms of ALDH1 in normal tissues

Acetaldehyde dehydrogenase (ALDH) is a randomly assembled tetramer in the body and contains 19 functional ALDH genes in the human genome (15), and ALDH1 introduced in this study is one of the more critical subgroups. The ALDH1 family consists of six human ALDH genes, including ALDH1A1, ALDH1A2, ALDH1A3, ALDH1B1, ALDH1L1, and ALDH1L2. The rat and mouse genomes contain an additional gene, ALDH1A7, which is 92% identical to the mouse ALDH1A1 (16). ALDH1 can not only be used as a marker of cancer stem cells but also plays an irreplaceable role in promoting physiological functions such as alcohol metabolism and synthesis of retinoic acid (RA).

In normal human stem cells, ALDH1 can irreversibly convert the retinal to RA in the cytoplasm. Then RA will be transferred to the nucleus, activating the retinoic acid receptor (RAR), retinoic acid X receptor (RXR), and nuclear hormone receptor peroxisome proliferator-activated receptor β/δ (PPARβ/δ) to regulate the transcriptional activity of more than 500 genes (17), which play an essential role in human development and maintain homeostasis of human organs.

### Mechanisms of ALDH1 in CSCs

**Figure 1:**
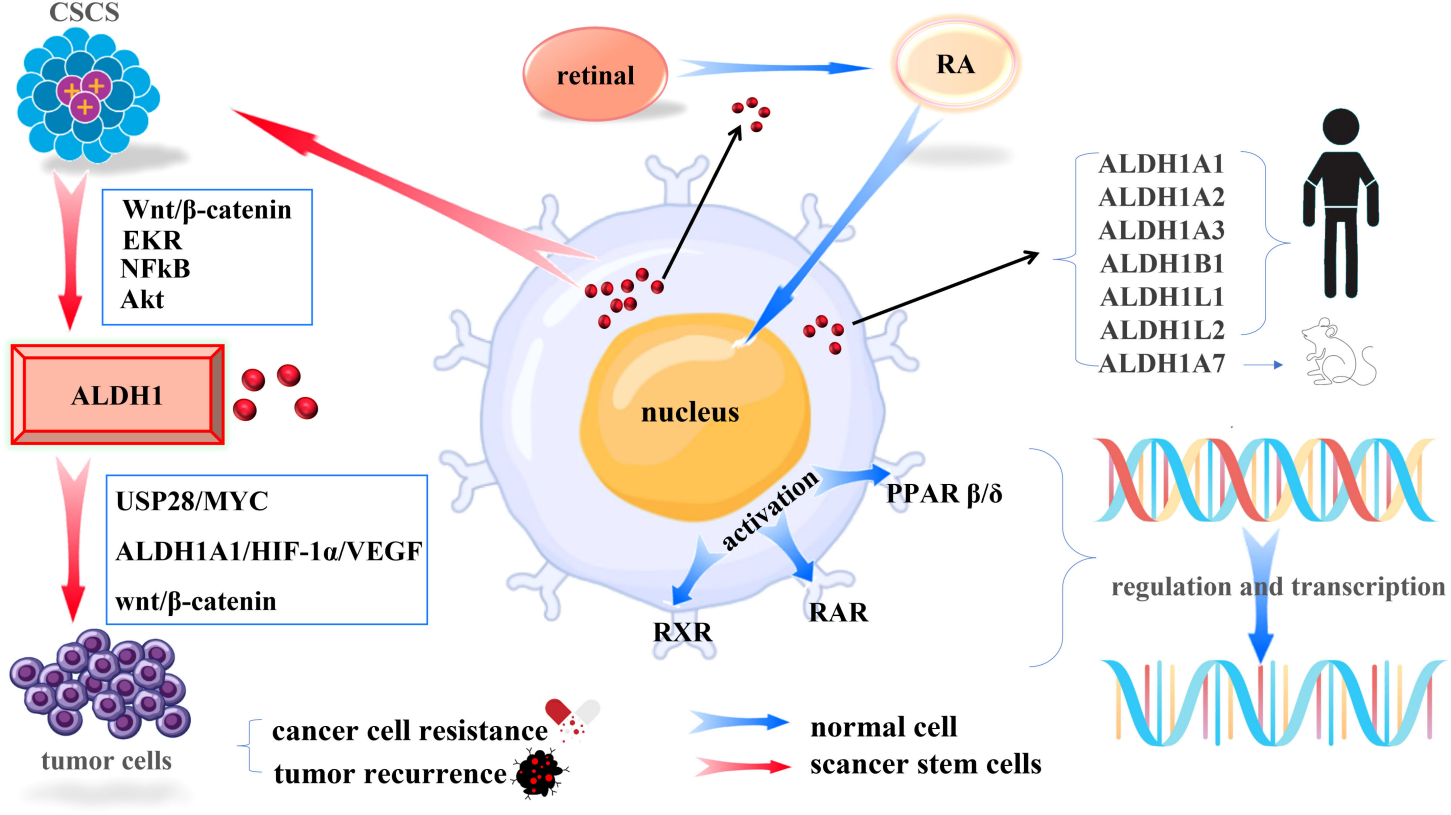
Mechanisms of ALDH1 in Cells. (Blue arrows indicate the mechanism of action of ALDH1 in normal tissues, red arrows indicate the mechanism of action of ALDH1 in cancer stem cells).

ALDH1 is considered a marker of CSCs, which can induce cancer by maintaining the characteristics of CSCs, modifying metabolism, and promoting DNA repair. ALDH1 is lowly expressed in normal tissues and highly expressed in cancer the expression level of ALDH1is a marker that distinguishes normal stem cells from cancer stem cells. Clinical research studies have found that the prognostic ratio of the ALDH1 gene family can be used as a robust poor predictor of various solid cancers, including breast cancer, colon cancer, esophageal squamous cell carcinoma, non-small cell lung cancer, ovarian cancer, and other cancers (18, 19). As a strong predictor, ALDH1 is also involved in deriving drug resistance in solid cancers. Although still controversial, it is undeniable that cancer cells with high ALDH activity and other stem cell-like characteristics are closely related to drug resistance and tumor recurrence. The underlying mechanism is currently unclear but may involve RA biosynthesis, scavenging of reactive oxygen species (ROS) and toxic aldehydes (20), USP28/MYC signaling pathway (21), ALDH1A1/HIF-1α/VEGF axis (22), wnt/β-catenin signaling pathway (23). As shown in **Figure 1**. From the above, we can boldly propose ALDH1 as a potential therapeutic target for solid cancer and provide a new ideas for further and more accurate prognosis and the development of new therapeutic targets.

## ALDH1 and various solid tumors

### ALDH1 and breast cancer

Breast cancer is the most common cancer in the world and the leading cause of cancer death in women. IARC estimates that by 2040, new breast cancer cases will exceed 3 million each year (1). Although endocrine therapy, radiotherapy, and chemotherapy have greatly improved the overall survival rate of breast cancer patients, the limitation of these treatment options is that they cannot target CSCs, leading to drug resistance and tumor recurrence, which is still incurable (24, 25). Elevated ALDH1 levels are associated with resistance to chemotherapy in breast cancer patients treated with a taxane-doxorubicin-cyclophosphamide regimen (26). ALDH1 contributes to normal and tumor stem cell differentiation and breast cancer invasion and metastasis are mediated by tumor cell subsets that exhibit stem cell-like featcharacteristics express ALDH1 (10). ALDH1 functions primarily by regulating vitamin A oxidation, and the expression level of ALDHI has become a marker for distinguishing normal stem cells from tumor stem cells in breast tissue (27, 28) and is significantly correlated with a poor prognosis (29–31). ALDH1A1 and ALDH1A3 can dramatically enhance ALDH1 activity and are associated with a poor prognosis in patients with breast cancer (32). The elimination of ALDH1A1, ALDH1A3 inhibits ALDH1 activity, increases chemosensitivity, and reverses chemoresistance in breast cancer (33).

Based on its enzymatic activity, ALDH1A1 reduces the intracellular pH of breast cancer cells and upregulates GMCSF by activating the TAK1-NFkB signaling pathway. It induces MDSC expansion, thus decreasing antitumor immunity and promoting breast cancer progression (34). ALDH1A1 also enhances the USP28/MYC signaling pathway to promote breast cancer stem cells by maintaining a local acid microenvironment (35). The breast cancer stemness marker ALDH1A1 activates ALDH1A1/HIF-1α/VEGF axis through retinoic acid conduction, upstream HIF-1α is activated, induces VEGF expression and release, and promotes tumor angiogenesis (22). ALDH1 activation and transcription are is mainly related to the MUC1-C/TWIST1/EMT pathway (36), MUC1-C→ERK→CEBPβ→ALDH1A1 pathway (20, 37), Nanog signaling (38), Wnt/β-catenin pathway (39), Notch, TGF-β pathway (40), SIRT1-PRRX1-KLF4-ALDH1 pathway (41), IL-6/STAT3/ALDH1 path (42) and so on.

Studies have shown that targeting or inhibiting ALDH1 can alleviate breast cancer. CAP targets ALDH1 breast CSCs by regulating AQP3-19Y-mediated ubiquitination of AQP3-5K and FOXO1 K48, which can improve therapeutic efficacy (43). Targeting ALDH1 in breast CSCs with ATRA or N,N-diethylaminobenzaldehyde (DEAB) combined with doxorubicin or paclitaxel therapy and radiation therapy significantly reduced tumor cell viability (38). The ALDH1 inhibitor disulfiram inhibited breast tumor growth and occurrence (34). Limonin (44), quercetin (45), and curcumin (46) inhibit breast cancer stem cells by downregulating ALDH1A1. ALDH1 may serve as a potential therapeutic target for breast CSCs (47), so it can provide a new therapeutic target and a basis for breast CSCs.

### ALDH1 and lung cancer

The mortality rate of lung cancer ranks first among malignant tumors worldwide (48). Although surgery and chemotherapy have a specice, particular effect on their treatment, the 5-year prognosis of patients is severe, mainly due to tumor metastasis and drug resistance (49–51). ALDH1, as a lung CSC marker (52, 53), is associated with a poor prognosis and resistanceto treatment in lung cancer patients (54, 55). High expression of ALDH1 is negatively correlated with patient survival (56). Targeting ALDH1 could be a new strategy to overcome drug resistance (51).

The study shows that ALDH1 promotes functional changes in the glutathione redox system and enhances chemosensitivity in non-small cell lung cancer (57). ALDH1A1 confers resistance to erlotinib by promoting a ROS-active carbonyl species metabolic pathway in lung adenocarcinoma (51). Lung adenocarcinoma cells overexpressing ALDHA1A1 can meet rapid growth of tumor cells or respond to drug stress through the Warburg effect (58). High expression of ALDH1 can be achieved by activating the MEK/ERK signaling pathway (59). S100A9 upregulates ALDH1A1 expression and activates the RA signaling pathway in lung cancer cells (60). Regulation and expression of ALDH1 in lung cancer are mainly related to TSPYL5 (61), STAT3 (62), SOX9 (63), β-catenin (64), MiR-34a/IL-629 (54), miR-326/GNB1 (65), RNAMACC1-AS143 (66), PFKFB346 (67) and other signals and pathways.

Several studies on the treatment of lung cancer have shown that treatment strategies that reduce ALDH1 or target ALDH1 can reduce chemotherapeutic drug resistance and malignant proliferation of lung cancer. The vitamin A/retinoic acid axis depletes ALDH1 positive CSCs and resensitizes drug resistant lung cancer cells to cisplatin (53). Targeting the s100A9-ALDH1A1-retinoic acid signaling pathway inhibits brain recurrence in EGFR-mutant lung cancer (60). Standard drugs for lung cancer treatment, cisplatin/gemcitabine,and menadione, reduced ALDH1 expression (68), while the elimination of ALDH1A1 significantly increased apoptosis and decreased resistance to cisplatin (69). Fat1 overexpression (70), aerobic exercise (71), nilotinib, erlotinib (72), all-trans retinoic acid (73), itraconazole (74), cryptotanshinone (55), CFTR31 (75), fluorescent interleukin (76), pomegranate (77), ginsenoside Rg3 (78), puerarin 6″-O-xyloside (79), glycolysis inhibitor PFK158 (67), globulin (80) can reduce expression of ALDH1 and drug resistance in lung cancer, mainly involved NF-κB pathway, Wnt pathway, Src-STAT3 signaling axis, Wnt/β-catenin/STAT3 axis, Akt/c-Myc signaling pathway. Therefore, in the treatment of lung cancer, targeting ALDH1 has broad prospects and should be further studied and explored.

### ALDH1 and colorectal cancer

Colorectal cancer (CRC) is the third most common cancer worldwide and the second leading cause of cancer-related death worldwide (81). Most CRC patients die from recurrence, distant metastasis, and chemotherapy resistance (82, 83), mainly due to a small subpopulation of cells within the tumor called CSCs. Increased expression of ALDH1 is associated with tumor progression and poorer outcomes in CRC patients (11). ALDH1 is abundant in tumor samples from patients with CRC (21, 84), which can be used as a particular marker for CSCs of colorectal cancer (85), mainly including ALDH1A1, ALDH1B1, and ALDH1A3, are associated with poor prognosis and resistance to chemotherapy in colorectal cancer (86–88).

ALDH1 plays a vital role in CRC and promotes the metastasis and proliferation of CRC stem cells through various classical pathways. Studies have suggested that the high expression of ALDH1 in CRC is related to the Wnt/β-catenin pathway (82, 89, 90), hsa_circ_0001806/miR-193a-5p/, COL1A1 axis (89), PI3K/AKT/mTOR signaling pathway (91), CXCL2/CXCR2 axis (92), miR-200-ZEB1/SANI2 axis (88). Additionally, ALDH1B1 may maintain the CSC phenotype and promote cancer cell growth by protecting cells from DNA damage (87). Its presence is closely related to the activation of Wnt/β-catenin (93). LncRNA NEAT1 increases H3K27ac by affecting chromatin remodeling, leading to increased levels of acetylation in the ALDH1 and c-Myc promoter regions to improve the stemness of colorectal cancer cells (83). p53 (94), P2X7R (95), and lncRNA B4GALT1-AS1 (96) can up-regulate ALDH1 expression in CRC.

In an effort to address the abnormal expression and related pathways of ALDH1 in colorectal cancer, targeting or inhibiting ALDH1 has become a new direction for treating colorectal cancer. The study proposes that physciosporin inhibits the stemness of colon cancer cells and the expression of ALDH1 through the Sonic Hedgehog and Notch signaling pathways (97). Similarly, silibinin down-regulates the cancer stemness marker ALDH1 by modulating the E-Cadherin/β-Catenin pathway (98). Furthermore, tumidulin reduces ALDH1 in CRC cells by inhibiting The Hh signaling pathway (99). Inhibiting of ALDH1A1 expression can down-regulate oxidative phosphorylation, mitochondrial function, the sirtuin signaling pathway, cholesterol biosynthesis, and the vitamin A (retinol) metabolism pathway (93). Numerous studies point to inhibition of DCLK1 (100), down-regulation of MUC1-C (101), down-regulation of ALDH1B1 (102), down-regulation of KDM2B (103), down-regulation of NEAT1 (83), polymethoxylated flavones (104), resveratrol (105), ALDH1A3 inhibitor (88), 5-fluorouracil (106), grape pomace (107), montelukast (108), celecoxib targeted therapy (109), puerarin (110), can both down-regulate the expression of ALDH1 in CRC and reduce cell migration, invasion, and chemotherapy resistance. Therefore, the potential value of targeting ALDH1 to improve the efficacy of standard treatment and thus prevent recurrence of colorectal cancer remains to be further investigated.

### ALDH1 and liver cancer

Liver cancer is the third largest cancer in the world and the leading cause of cancer-related death (111). Liver cancer has a high degree of malignancy and is easy to metastasize, and most patients are in the middle and late stages when diagnosed (112). Most patients with advanced liver cancer are treated with chemotherapy, but are, incredibly prone to drug resistance, leading to failure of treatment, mainly related to liver CSCs (113). ALDH1 is considered a marker of liver cancer stem cells, and the high expression of ALDH1A1 is closely related to recurrence of liver cancer (114). Studies have found that PFKP can promote reverse transcriptional activation of β-catenin, leading to the expression of ALDH1 in liver cancer stem (115). Abnormally expressed PDK1 can covalently bind to the inactive ALDH1A1 apoenzyme (apoALDH1A1), forming the catalytically active ALDH1A1 holoenzyme (holoALDH1A1), thus activating ALDH1, leading to desensitization of liver cancer to radiation therapy (116). LncRNASNHG5 promotes hepatocellular carcinoma proliferation and tumor stem cell-like ALDH1 properties by regulating the UPF1 and Wnt signaling pathways (117). LncRNA LINC00460 regulates liver cancer cell proliferation by targeting the miR-503-5p/miR-654-3p/TCP1 axis (118). Long noncoding RNA MACC1-AS1 promotes ALDH1 in stem cells from hepatocellular carcinoma by antagonizing miR-145 (119). Carboxypeptidase A4 upregulates ALDH1 expression and promotes proliferation of hepatocellular carcinoma (120).

Studies have found that inhibiting or targeting ALDH1 liver cancer stem cells can reduce cancer proliferation and resistance to treatment. Silencing PFKP can inhibit the liver cancer stemness marker ALDH1 (121). Silencing shRNA-mediated MALAT1 significantly inhibited ALDH1 activity, partly due to the inhibition of the MALAT1/Wnt/β-Catenin pathway (122). STARD13 overexpression can reduce ALDH1 activity and improve 5-FU sensitivity in liver cancer, which is positively correlated with a good prognosis (123). Limonin reduces cell quiescence and reduces ALDH1 stem in liver cancer cells by activating PI3K/Akt signaling (124). UTI, inhibits Wnt/β-catenin signaling and attenuates the ALDH1-sensitivity of the liver cancer stem to FU (113). The box protein FBXO11 reduces ALDH1 activity and hepatocellular carcinoma stemness by promoting ubiquitin-mediated inhibition of snail degradation (125). Overexpression of TRPV2 minimizes the liver cancer stem cell marker ALDH1 (126). Therefore, targeting or inhibiting ALDH1 in liver cancer can enhance chemosensitivity, and provide potential novel therapeutic strategies and new insights for developing new therapeutic targets for liver cancer.

### ALDH1 and gastric cancer

Gastric cancer (GC) ranks fifth in incidence (5) and is one of the leading causes of cancer-related deaths. GC is usually diagnosed at an advanced stage when the tumor is inoperable and only chemotherapy may be a helpful method (127). Although traditional chemotherapy can significantly improve the survival rate of gastric cancer patients, chemotherapy alone is still very limited and has reached a bottleneck (128). Therefore, it is urgent to find new targets for molecularly targeted therapy of GC. GCs with histological diversity show a poor prognosis and characteristic expression of the cancer stem cell-associated molecule ALDH1 (129). High ALDH1 expression is associated with poor prognosis in gastric neuroendocrine carcinoma (130, 131). The presentation of ALDH1 in gastric cancer tissue was significantly higher than in normal tissue, The manifestation of ALDH1 was significantly correlated with tumor grade, tumor stage, lymph node metastasis, tumor metastasis stage, and overall survival of patients (132).

The present results suggest that ALDH1 overexpression may be involved in the occurrence, invasion, and metastasis of GAC, leading to a poor prognosis (132). High expression of ALDH1 in GC cells improves stem cell properties and antagonizes the action of macrophages, thus affecting cell viability, anti-apoptosis, invasion, migration, and cloning ability. ALDH1 is positively correlated with helicobacter pylori infection. When ALDH1 is overexpressed, it mainly restores the reduction of drug resistance caused by miR-625 overexpression. It is involved in the regulation of various genes and proteins in the process of GC occurrence and development (5). Furthermore, GC cells with high expression of ALDH1 can evade the deadly effect of macrophages by antagonizing tumor necrosis factor α and other effector molecules secreted by macrophages and increase tumor proliferation and invasion ability (133). Studies have shown that overexpression of TAZ (134), upregulation of A1 in cancer-testes (135), and knockdown of HMGA2 (136) can promote the high expression of ALDH1 in the GC and tumor growth.

With extensive research on ALDH1 in recent years, it has been found that targeting drugs can inhibit the growth of GC and reverse drug resistance by inhibiting ALDH1expression. All-trans retinoic acid appears to inhibit tumor growth, target gastric CSCs, and reduce ALDH1 expression (137). ALDH1A1 silencing inhibits cell viability by modulating Wnt signaling in the migration and invasion of MKN-45 cells. Small interference RNA, high expression of Ror β, salinomycin, and FoxM1 siRNA transfection inhibited the expression and invasive capacity of ALDH1 in GC (5). Silencing miR-95 or overexpressing a miR-95 inhibitor (dual specificity phosphatase 5) inhibited ALDH1 expression in GC cells (138). BRD4 promotes the stemness of gastric cancer cells and reduces ALDH1 activity by attenuating the mir-216a-3p-mediated inhibition of the Wnt/β-catenin signaling pathway (134). Therefore, ALDH1 may be a new target for related tumor suppressors and stem cells.

### ALDH1 and cervical cancer

Cervical cancer is the fourth most common cancer and the most common gynecological malignancy (139), of which squamous cell carcinoma (SCC) is the most common type, accounting for approximately 80% of all cervical cancers (140). Treatment of advanced cervical cancer includes surgery, chemotherapy, and radiation therapy, but cervical cancer mortality remains high (141), mainly related to CSCs (142). Studies have suggested that ALDH1 can be used as a cervical CSC marker (143, 144), which can lead to resistance to chemotherapy and is closely related to a poor prognosis (145).

Studies have shown that ALDH1 is the target of miR-222, and miR-222 can bind to the 3’untranslated seed region of ALDH1 mRNA to regulate its expression, resulting in an elevated expression level of ALDH1 in cervical cancer (146). At the same time, studies have indicated that ALDH1 expression in cervical cancer is closely related to the Erk1/2 and Akt signaling pathways (147). Hypoxia promotes ALDH-1 expression in radioresistant cells, and ALDH-1-positive cells promote radioresistance in cervical cancer by preferentially activating DNA damage checkpoint responses and increasing DNA repair capacity (143). ALDH1 expression is associated with higher cell proliferation, spheroid formation, migration, and tumor incidence in cervical cancer cells, which exhibit chemo and radioresistance (148).

In recent years, some achievements have been made in inhibitory drugs or targeted therapy for ALDH1. For example, PM01183 (149), zoledronic acid (147), and limonin (150) can inhibit the activity of the cervical CSC marker ALDH1, weaken the stemness of cervical cancer cells, and waste their chemoresistance. Therefore, ALDH1 may be a new target for cervical cancer, which may provide a new and promising strategy for anticancer therapy, which is worthy of further exploration.

### ALDH1 and esophageal cancer

Esophageal cancer is the sixth most common cause of cancer death worldwide (151), and 90% of esophageal cancers are esophageal squamous cell carcinoma (ESCC), which has an inferior, an abysmal prognosis and high mortality (152). Esophageal cancer has a low 5-year survival rate and lacks effective therapeutic targets (153). In ESCC, ALDH1 is a more reliable CSC marker, and high expression of ALDH1 is associated with poor differentiation from ESCC and poor prognosis (154, 155).

Studies have shown that ALDH1A1 can activate AKT, interact with β-catenin, and start the wnt/β-catenin signaling pathway to maintain the CSC properties of ESCC (23). The study suggested that the high expression of ALDH1A1 is also associated with the promoting EMT in ESCC (156). ALDH1 plays a vita role in tumor aggressiveness and is associated with the pro-tumor microenvironment of esophageal cancer, mainly involving the IL-6/STAT3 pathway and factors such as p-STAT3, MDSC2 (12). Similarly, ALDH1A1 knockdown or the use of the small molecule inhibitor NCT-501 reduced the level of AKT phosphorylationin in A549/DDP cells and inhibited the AKT-β-catenin signaling pathway (23). Animal experiments demonstrated that COX-2 inhibition reduced ALDH1 and IL-6 expression levels, attenuated MDSC recruitment, and subsequently slowed esophageal tumors (12). Tranilast significantly reduced the strong expression of ALDH1A1 in TE8 cells (157). Additionally, CA3 and LEE011 can jointly inhibit the YAP1 and CDK6 pathways, significantly reduce the growth of esophageal cancer cells and cancer stem cell ALDH1, sensitize cells to radiation, and show strong antitumor effects *in vivo* (158). Therefore, ALDH1 is expected to become a new direction for esophageal cancer stem cell research.

### ALDH1 and ovarian cancer

Ovarian cancer (OC) is the most lethal gynecological malignancy and ranks first in cancer mortality among female malignancies (159). Epithelial ovarian cancer (EOC) accounts for 95% of ovarian malignancies, and is the most common OC (160). The prognosis of OC treatment is unsatisfactory mainly, probably due to the presence of ovarian cancer stem cells (OCSC) and chemoresistance (7, 160). In OC, high ALDH1 is a hallmark of OCSC (161–163) and is strongly associated with poor prognosis, and resistance to chemotherapy (164–166).

Studies support that OC patients with high expression of the ALDH1A1 stemness gene have an attenuated response to platinum-containing chemotherapy (167) and that ALDH1A1 may be involved in acquired resistance to cisplatin through upregulation of NEK-2 in OC (166). Platinum-induced secretion of IL-6 from cancer-associated fibroblasts in the tumor microenvironment promotes the enrichment of OCSC in residual tumors after chemotherapy through activation of STAT3 and up-regulation of ALDH1A1 expression (45). ALDH1 activity has also been reported to be positively regulated by the BET family protein BRD4, which is capable of to up-regulate ALDH1A1 transcription through super-enhancer elements (168). AhR can also mediate OC progression, a stem ALDH1 signature, by activating PI3K/Akt, Wnt/β-catenin, and EMT (169). Furthermore, the expression and activity of ALDH1A1 can be regulated by β-Catenin, the EZH2 enhancer, and the bromodomain and extra terminal (BET) protein family (170).

Recent studies have suggested that customized therapy targeting ALDH1 can reduce resistance to chemotherapy and improve the survival rate of OC. ALDH1A1 knockdown can reverse the resistance to OC chemotherapy (168). Dual inhibition of Src and MEK reduces OC growth and targets ALDH1 (171). Consistent with these, ALDH1 inhibition effectively blocks the proliferation and survival of OC spheroids (167), and aldehyde dehydrogenase inhibitors promote OC DNA damage (172). IL-6-Nab combined with HMA completely eradicated OCSCs, and this combination blocked IL-6/IL6-R/pSTAT3-mediated ALDH1A1 expression, providing a strategy for tumor recurrence after chemotherapy (173). NAMPT inhibitors and 4-MU treatment reduce ALDH1 protein expression, which reduces chemoresistance and OC cell growth (174, 175). BET inhibitors inhibit ALDH activity by abrogating BRD4-mediated expression of ALDH1A (176). DDB2 inhibits OC cell dedifferentiation by inhibiting ALDH1A1 (170). A selective inhibitor of the ALDH1A family (ALDH1Ai) reduces tumor-initiating capacity and chemoresistance (177). With the continuous in-depth understanding of ALDH1 and drug resistance mechanisms in OC, the development of drugs targeting ALDH1 is expected to become a new direction for treating OC.

### ALDH1 and head and neck carcinomas

Head and neck carcinomas (HNCs) are the sixth most common cancer worldwide (178), which are characterized by the unregulated growth of tumor cells in various parts of the head and neck, such as the buccal mucosa, floor of the mouth, tongue, oropharynx, hypopharynx, esophagus, nasopharynx, and salivary glands (179), and more than 90% of HNCs are squamous cell carcinomas (HNSCC) (180). HNCs have poor survival, and poor prognosis (181), and CSCs have been attributed to poor treatment outcomes and survival in HNSCC (13). ALDH1 is associated with tumor malignancy and self-renewal properties of stem cells in HNCs (182) and has been associated with poorer prognosis and failure of chemoradiotherapy in HNCs (13, 183). ALDH1 acts as a stem cell marker for HNCs such as oral cancer (184), eyelid sebaceous gland carcinoma (185), benign epithelial odontogenic lesions (186), salivary gland tumor (187, 188), lip cancer (189), sublingual adenoma (190).

Studies have shown that in HNCs, ALDH1 is mainly regulated by retinoic acid compounds and other oncogenic pathways such as MUC1-C/ERK and WNT/β-catenin (191), but also by the AKT signaling pathway (192, 193). ALDH1A1 increases TUBB3 expression, which down-regulates PTEN and promotes cell proliferation, migration, and invasion (194). Inhibition of miR-30a and miR-379 upregulates DNMT3B expression, which leading to hypermethylation of the ALDH1A gene, and promotes oncogenic activity (195, 196). Expression of ATAD2 (154), VIM, or ZEB2 (197) promotes ALDH1 expression. Similarly, the ovatodiolide can inhibit ALDH1 activity and stemness properties by inhibiting JAK2/STAT3/JARID1B signaling (198). The soy isoflavone genistein hinders stem cells in HNCs by activating the miR-34a/RTCB axis and reducing ALDH1 activity (199). Butylene phthalide (200), honokiol (201), elimination of long noncoding RNA MACC1-AS1 (202), resveratrol (203), can reduce ALDH1 activity and inhibit the stemness of HNCs. Therefore, ALDH1 is a possible drug target in HNCs.

### ALDH1 and other cancer

In recent years, a large number of studies have shown that ALDH1 is also closely related to other solid tumors.The overexpression of ALDH1 is associated with a poor prognosis and malignant tumor development, such as melanoma (204), glioma (205), prostate cancer (206, 207), endometrial cancer (6, 208), bladder cancer (209), Osteosarcoma (210), pancreatic cancer (211, 212), kidney cancer (213), etc. ALDH1, overexpressed in melanoma, promotes tumor angiogenesis mainly by activating IL-8/Notch signaling (214). Additionally, ALDH1A1 can metabolize retinal RA, thus activating RAR-mediated transcription of downstream targets, such as TUBB3, in bladder cancer cells (209). ALDH1A3 promotes pancreatic cancer metastasis through its metabolic effects on glucose metabolism, and PPARγ and its downstream PI3K/AKT/mTOR signaling pathway may be involved in this process (215). Furthermore, the expression of ALDH1 in these solid tumors is also affected by Notch1 signaling (216), ILK signaling (217), Hippo signaling (218), miR-761 (219), ARID1A deficiency (220), IL17 (221), etc.

Similarly, inhibition or targeting of ALDH1 can alleviate the progression of these solid tumors, such as acid sensor ASIC1a (222), depletion of TP73-AS1 (223), eprinomectin (224), silencing of YAP (218), MicroRNA-487b-3p (225), miR-199 (226), CTSB knockdown (227). Therefore, ALDH1 is expected to become a corresponding therapeutic target for the solid tumors mentioned above.

## Prospective

By combing all articles on ALDH1 and solid tumors in the last five years, it is found that ALDH1 can be used as a marker of tumor stem cells, expressed in a variety of malignant tumor tissues; the expression level of ALDH1 is a marker that distinguishes normal stem cells from cancer stem cells. ALDH1 can maintain stem cell characteristics and lead to drug resistance, which is the key to tumor recurrence and is difficult to cure. Preclinical studies have shown that ALDH1 plays an essential role in the occurrence, invasion, and metastasis of different cancers through various pathways (20) (220) (22) (23). In recent years, many scholars have studied ALDH1 inhibitors and targeting ALDH1, but there is no clinical evidence on the efficacy and safety of their inhibition in solid tumors. Although none of these newer compounds has entered clinical trials and are still in the early stages of ALDH1-targeted therapy development, they have shown promising effects and have an encouraging future for ALDH1-targeted drugs. Therefore, further research will be crucial to make ALDH1 a therapeutic target.

## Author contributions

YW and YL drafted the manuscript. All authors contributed to the article and approved the submitted version.

## Funding

This study was supported by the following grants: Hubei Department of Education Science Research project (grant no. Q20192802) for QW, National Natural Science Foundation of China Youth Science Foundation project (grant no. 81902937) for YS.

## Acknowledgments

The authors thank the School of Basic Medicine Sciences, Xianning Medical College, Hubei University of Science and Technology.

## Conflict of interest

The authors declare that the research was conducted in the absence of any commercial or financial relationships that could be construed as a potential conflict of interest.

## Publisher’s note

All claims expressed in this article are solely those of the authors and do not necessarily represent those of their affiliated organizations, or those of the publisher, the editors and the reviewers. Any product that may be evaluated in this article, or claim that may be made by its manufacturer, is not guaranteed or endorsed by the publisher.

## Abbreviation

CSCs, cancer stem cells; ALDH1, acetaldehyde dehydrogenase 1; RA, retinoic acid; RAR, retinoic acid receptor; RXR, retinoic acid X receptor; PPAR/β/δ, peroxisome proliferator-activated receptor β/δ; ROS, reactive oxygen species; ATRA, all-trans retinoic acid; DEAB, N,N-diethylaminobenzaldehyde; CRC, colorectal cancer; GC, gastric cancer; ESCC, esophageal squamous cell carcinoma; OC, ovarian cancer; EOC, Epithelial ovarian cancer; OCSC, ovarian cancer stem cells; BET, bromodomain and extraterminal; HNSCC, Headneck squamous cell carcinoma.
